# High Efficiency Fabrication of Chitosan Composite Nanofibers with Uniform Morphology via Centrifugal Spinning

**DOI:** 10.3390/polym11101550

**Published:** 2019-09-24

**Authors:** Zhen Li, Shunqi Mei, Yajie Dong, Fenghua She, Lingxue Kong

**Affiliations:** 1 School of Mechanical Engineering and Automation, Wuhan Textile University, Wuhan 430073, China; zkj@deakin.edu.au (Z.L.); dongy@deakin.edu.au (Y.D.); 2 Institute for Frontier Materials, Deakin University, Geelong, VIC 3216, Australia; mary.she@deakin.edu.au; 3 Hubei Key Laboratory of Digital Textile Equipment, Wuhan 430073, China

**Keywords:** carboxylated chitosan, centrifugal spinning, nanofiber diameter, empirical model

## Abstract

While electrospinning has been widely employed to spin nanofibers, its low production rate has limited its potential for industrial applications. Comparing with electrospinning, centrifugal spinning technology is a prospective method to fabricate nanofibers with high productivity. In the current study, key parameters of the centrifugal spinning system, including concentration, rotational speed, nozzle diameter and nozzle length, were studied to control fiber diameter. An empirical model was established to determine the final diameters of nanofibers via controlling various parameters of the centrifugal spinning process. The empirical model was validated via fabrication of carboxylated chitosan (CCS) and polyethylene oxide (PEO) composite nanofibers. DSC and TGA illustrated that the thermal properties of CCS/PEO nanofibers were stable, while FTIR-ATR indicated that the chemical structures of CCS and PEO were unchanged during composite fabrication. The empirical model could provide an insight into the fabrication of nanofibers with desired uniform diameters as potential biomedical materials. This study demonstrated that centrifugal spinning could be an alternative method for the fabrication of uniform nanofibers with high yield.

## 1. Introduction

Chitosan is a natural polymer that has been widely studied for tissue engineering, due to its excellent biodegradable properties, antibacterial abilities and hemostasis properties [1,2,3,4]. However, toxic or dilute acidic solutions, such as chloroform [5,6], acetic acid [7], or 1,1,1,3,3,3,-hexafluoro-2-propanol (HFIP) [8] were used as solvents to make chitosan solution. Those produced chitosan nanofibers could be toxic because of solvent residues [9]. Therefore, it is imperative to develop new materials and processes that don’t use any toxic components. CCS is a water-soluble chitosan derivative, which can be a better option for potential wound healing activities, due to no toxic dilute acidic being used [10]. In addition, only deionized water (DI water) was used to dissolve CCS, so as to produce CCS composite nanofibers as bionic scaffold for skin tissue regeneration and drug delivery [1,2,11]. 

Spinning pure or high percentage of continuous bead-free chitosan nanofibers is extremely difficult [12]. Polyethylene oxide (PEO) could usually be used to improve the dimensional stability of chitosan composite nanofiber meshes [13], due to it being biodegradable, hydrophilic and non-toxic [14,15,16]. However, maintaining a high percentage of chitosan in nanofiber scaffold is also important in tissue regeneration as the antibacterial properties of composite nanofibers mainly rely on chitosan. Zhou et al. [17] fabricated composite nanofibers with 40% CCS for tissue engineering.

Diameter of fibers plays a significant role in tissue engineering, because the natural collagen nanofibers in tissues are in the range of 50–500 nm [18]. It indicates that continuous bead-free nanofibers should be produced with fiber diameter in this range, so as to maximize the effect of tissue regeneration [19]. However, Ko et al. [20] and Bienek et al. [21] only fabricated chitosan composite nanofibers with fiber diameter from 634–913 nm and 981–1139 nm, respectively which are much larger than the ideal range. Besides, aligned nanofibers have a huge potential to guide cell growth for muscle and nerve fiber repair due to their unique electrical and mechanical properties [22]. Therefore, it is important to stably fabricate uniform, aligned and continuous bead-free nanofibers that mimic the natural tissue fibers for the repairing of damaged tissue structure.

**Figure 1:**
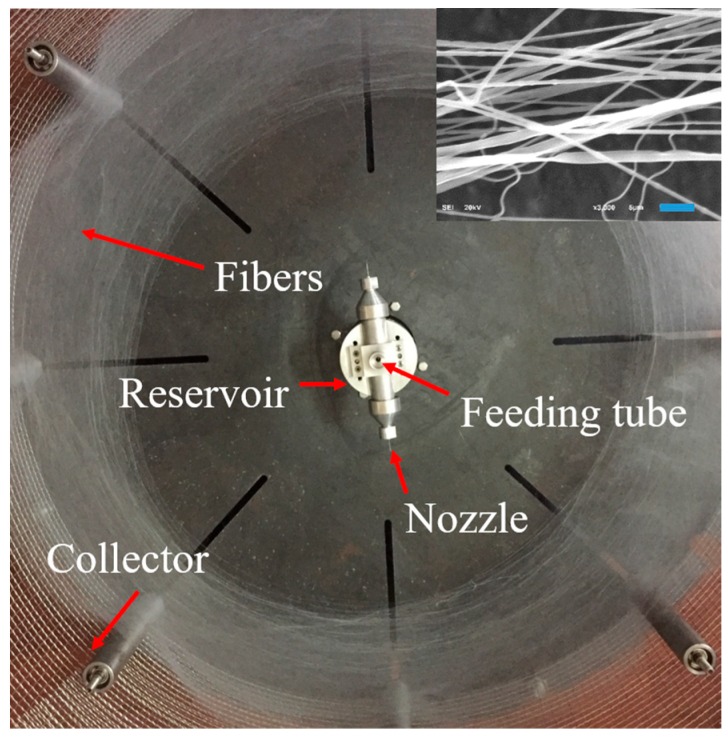
PEO nanofibers fabricated from centrifugal spinning machine via 6 w/v% PEO solution with 4000 rpm, 0.15 mm nozzle diameter and 13 mm nozzle length. The inset is the SEM image of the obtained fibers, the scale bar is 5 µm.

Currently, electrospinning is the most popular method to produce nanofibers with uniform diameter distribution [23,24,25,26]. However, electrospinning still has some serious disadvantages, such as low spinning rate (less than 1 mL/h) and high energy consumption [27,28]. Hence, needleless electrospinning was designed and patented to improve the output of nanofibers, while, the alignment of uniformity of nanofibers via needleless electrospinning is very difficult to control [29]. Alternatively, centrifugal spinning is an ideal method to combine the advantages of needle and needleless electrospinnings, so as to produce uniform and aligned nanofibers with high efficiency [27,30,31]. In centrifugal spinning system, the centrifugal force overcomes surface tension force of polymer solution or melt, then, the solution or melt is ejected from nozzles. After solvent evaporation, the fibers are formed and collected on the collector, as shown in Figure 1. The diameter and alignment of fibers could be controlled by changing various parameters. Concentrations, rotation speeds and nozzle diameters are frequently studied, as they have a significant impact on nanofiber quality [31,32,33,34]. 

Fiber diameter could be controlled by changing the parameters of centrifugal spinning process [27]. However, it is not easy to make various weight ratios of CCS/PEO solutions to produce nanofibers with the same fiber diameter distribution (average fiber diameter less than 500 nm). Therefore, before spinning CCS/PEO nanofibers, in order to simplify the spinning process, pure PEO solutions were used to fabricate different nanofibers by changing the key parameters, including concentration, nozzle diameter, nozzle length and rotational speed (in Table 1). According to the data, an empirical model was built to predict the spinning of fibers with average diameters less than 500 nm with different ratios of CCS/PEO solutions. In this paper, high-yield centrifugal spinning system was utilized to produce uniform and aligned polymer nanofibers, so as to overcome the drawbacks of electrospinning. The capability of fabricating uniform and aligned chitosan composite nanofibers via high-efficiency centrifugal spinning method could accelerate the industrialization of nanofibers for tissue engineering.

## 2. Experimental

### 2.1. Materials

Carboxylated chitosan (low molecular weight) and polyethylene oxide (MW ~ 1,000,000) were purchased from Aladdin Industrial Corporation in Shanghai, China, which were used as received without further modifications. Deionized water was produced in the laboratory.

### 2.2. Fabrication of Pure PEO Nanofibers 

PEO solutions were prepared by dissolving PEO into deionized water. Four different parameters were varied to produce PEO nanofibers via a dedicated designed centrifugal spinning device [35], respectively: concentrations: 5–8 w/v%; rotational speeds: 3000–5000 rpm; nozzle diameters: 0.15 mm, 0.1 mm and 0.06 mm; nozzle length: 6 mm, 13 mm and 25 mm. All these experiments were performed at room temperature and the collection distance was 30 cm. 

### 2.3. Fabrication of CCS/PEO Nanofibers with High Content of Chitosan

14 w/v% CCS solution and 7 w/v% PEO solution were prepared by dissolving them into deionized water, respectively. Then, the volume of CCS solution and PEO solution were mixed with ratios of 2:1, 1:1 and 1:2. The ratio of CCS to PEO, r, could be described as $r=C_{C}\times V_{C}/C_{P}\times V_{P}$, where $C_{C}$ and $V_{C}$ are concentration and volume of CCS, $C_{P}$ and $V_{P}$ are concentration and volume of PEO. Therefore, the ratio of CCS to PEO in three solutions were 4:1, 2:1 and 1:1, respectively. It assumed that all the solvents will evaporate after fiber solidification, therefore, the contents of CCS in the three samples of fibers were 80%, 66.7% and 50%, respectively. 

### 2.4. Viscosity Measurements

AR-2000ex rheometer (TA Instruments) was used to measure the viscosity of PEO solutions (5–7 w/v%), CCS solution (14 w/v%) and CCS/PEO compound solutions with various ratios. A parallel plate (40 mm diameter) was applied to test each sample with a gap in 500 μm. The viscosity of polymer solutions was measured with increasing shear rate from 0.1/s to 1000/s at room temperature [30]. The zero-shear viscosity of each solution was analyzed by TA rheology analysis software.

### 2.5. Fiber Characterization

Fiber morphologies and diameters were examined by scanning electron microscopy (SEM, JSM-6510LV, JEOL, Tokyo, Japan). Before SEM testing, the obtained polymer fibers were coated with Au by sputter coater (JFC-1600, JEOL, Tokyo, Japan). The diameters and the alignment of the polymer fibers were measured by ImageJ software (National Institutes of Health, Bethesda, MD, USA). For each of fiber specimen, 10 SEM images were taken and 100 counts of fibers were measured so as to present an objective statistical analysis of fiber morphology and diameter. Fourier transform infrared spectroscopy with a universal attenuated total reflection (FTIR-ATR, Vertex 70, Bruker, Karlsruhe, Germany) was used to test chemical characteristics. All CCS/PEO nanofibers were tested at wavelengths in the range of 4500–600/cm and a resolution of 1/cm with 64 scans. The thermal properties of fibers were tested by differential scanning calorimetric (DSC, 204F1, Netzsch, Selb, Germany) and thermogravimetric analysis (TGA, 209F1, Netzsch, Selb, Germany). Around 5 mg polymer fibers were used for each sample. In DSC test, the temperature increased from 30 °C to 250 °C with a heating rate of 10 °C/min. In TGA measurement, experiments were conducted over a temperature range from 10 °C to 800 °C with a heating rate of 10 °C/min. The mechanical test of CCS/PEO nanofibers was performed via an Instron 5943 ( Instron, MA, USA) with a 50 N load cell. The nanofibers were twisted into a yarn with 0.5 mm diameter and cut into 1 cm long, and set the extension rate at 2 mm/min. At least three specimens for each composite nanofibers were tested. Young’s modulus and failure work were calculated from the recorded stress-strain curve.

## 3. Results and Discussion 

### 3.1. Building Empirical Model 

**Figure 2:**
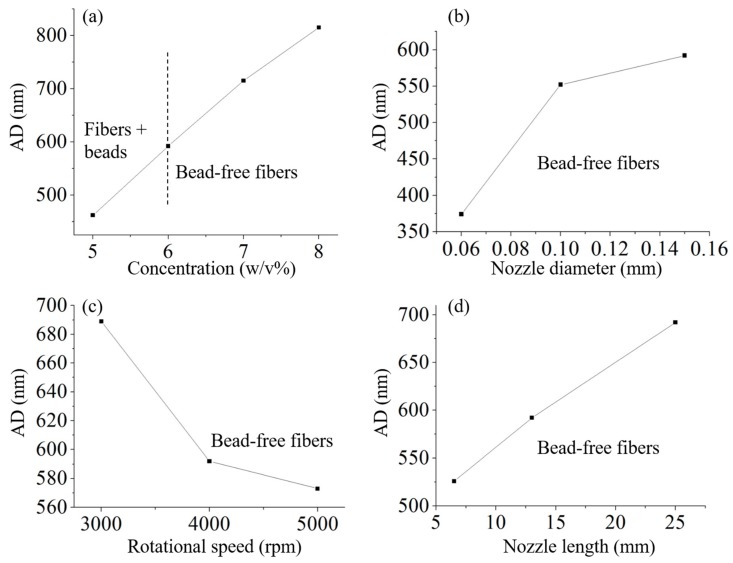
The average fiber diameter (AD) of collected nanofibers via different key parameters. (**a**) nozzle diameter: 0.15 mm, rotational speed: 4000 rpm, nozzle length: 13 mm; (**b**) rotational speed: 4000 rpm, concentration: 6 w/v%, nozzle length: 13 mm; (**c**) concentration: 6 w/v%, nozzle diameter: 0.15 mm, nozzle length: 13 mm; (**d**) nozzle diameter: 0.15 mm, rotational speed: 4000 rpm, concentration: 6 w/v%.

Formation of nanofiber is not only influenced by intrinsic material properties, but also effected by operational conditions [27]. Intrinsic material properties, which mainly include viscosity, concentration, molecular structure, molecular weight and surface tension [36,37,38], have a vital influence on the morphology and structure of nanofibers. However, for a certain polymer, solution viscosity can be easily controlled by changing polymer concentration and maintaining other parameters unchanged [39]. Operational conditions involve many factors, but rotational speed and nozzle diameter are the parameters most frequently investigated [40,41]. The influence of nozzle length was also involved, because it affected jet velocity and Capillary number [42]. Therefore, four different parameters were controlled to produce PEO nanofibers and investigate the relationship between fiber diameter and those parameters, as shown in Figure 2. It illustrated that fiber diameter increased with increasing concentration, nozzle diameter and nozzle length, respectively, while the fiber diameter decreased with increasing rotational speed. Specifically, higher concentration leads to larger viscosity and longer stress relaxation time, so as to restrict jet stretching and thinning [38]. Smaller nozzle diameter limited flow rate of solution, reduced jet radius at initial time and improved the ratio of surface and volume, comparing with larger nozzle diameter. Hence, thinner jet leads to faster solvent evaporation and thinner fiber could be collected [31]. Increasing rotational speed resulted in faster jet velocity and larger air flow; then, both jet stretching speed and solvent evaporation rate increased; finally, fiber could be thinner because jet can be stretched thinner and more solvent evaporated before solidification [37]. Nozzle length has been rarely studied, but is still an important parameter in centrifugal spinning system. Since the nozzle diameter is very small, there is a capillary effect existing during nanofiber fabrication. The jet has to overcome capillary effect of the nozzle, before it is ejected from nozzle. Therefore, *Ca* increased with increasing nozzle length. As longer nozzle length led to a slower jet velocity at initial time, both jet stretching speed and solvent evaporation rate decreased, resulting in larger fiber diameter. Besides, the interrelation of these parameters also impacts the final fiber diameter. For instance, when concentration was lower than critical concentration (6 w/v%), no continuous bead-free fibers could be formed, regardless of changing any other parameters, due to polymer chain entanglement being not sufficient against the jet breakup at the jet-extension stage [38]. Therefore, fiber diameter increased with increasing concentration, nozzle diameter and nozzle length, while decreased as rotational speed increased. The formation of nanofibers could be described by a group of dimensionless parameters, including Reynolds number ($R_{e}$), Weber number ($W_{e}$), Capillary number ($C_{a}$) and Weissenberg number (*W_i_*) [43]. These dimensionless parameters only described the jet trajectory and fiber formation, but could not predict final diameter of the fibers [44]. As these dimensionless parameters only contained intrinsic solution properties, other key parameters (such as rotational speed, nozzle diameter and nozzle length) were excluded.

**Figure 3:**
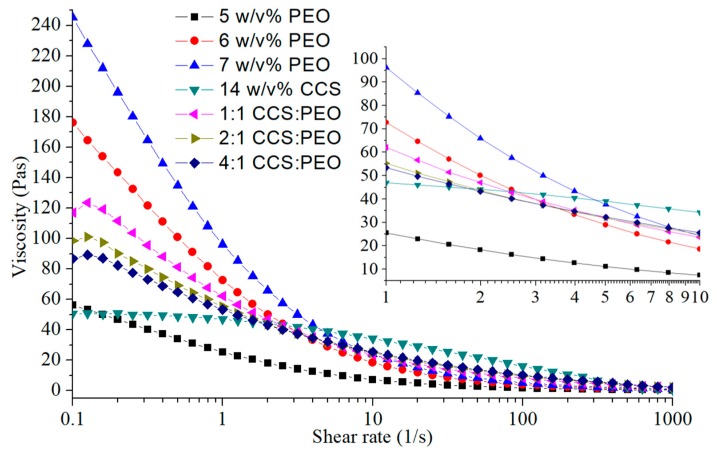
The viscosities of various polymer solutions.

Generally, polymer solution with high viscosities is a non-Newtonian liquid [45], but it has also been demonstrated that the polymer solution can be described as a Newtonian fluid [10,46,47,48,49,50] or a near-Newtonian fluid [31]. Figure 3 indicated that when the shear rate is over 100/s, the viscosities of polymer solutions changed linearly with shear rate. Polymer solution in centrifugal spinning could be considered as Newtonian fluid at high shear rate. Zhang et al. [27] reported that the thinning jet in centrifugal spinning process can be described as a Newtonian fluid, due to it being influenced by two inertial body forces (centrifugal force and Coriolis force). To produce bead-free fibers (as shown in Figure 1 and Figure 2), the jet relaxation process should be faster than the jet breakup time, and the solution should behave like a Newtonian fluid [39]. 

The dimensionless parameters had not been used to describe the thinning of filament, until Tripathi et al. [51] defined a processability parameter (P), and $h_{m}\eta /\gamma \equiv P=S_{h}C_{a}/R_{e}S_{c}$, where $h_{m}$ is the mass transfer coefficient of solvent, η is the viscosity, γ is the surface tension, $S_{h}$ is Sherwood number and $S_{c}$ is Schmidt number. Based on the processability parameter, Ren et al. [39] introduced another dimensionless number, elastic processability number, to predict final fiber diameter, $P_{e}=\lambda h_{m}/R_{0}$, where λ is jet thinning time scale, $h_{m}$ is the mass transfer coefficient of solvent and $R_{0}$ is the initial fiber radius. In this formula, $R_{0}$ could be seen as the nozzle diameter because the initial fiber radius is the radius of nozzle diameter. Therefore, the solution viscosity and nozzle diameter could be described by $P_{e}$. However, the final fiber diameter is not only determined by solution viscosity and nozzle diameter. The rotational speed and turning radius of nozzle (*L*) are crucial for centrifugal spinning system, because they are explicitly related to the centrifugal force, nozzle velocity and jet velocity at initial time. Therefore, the expected final fiber diameter could be described by a semi-analytical empirical model:

(1) $$D_{f}\sim \frac{R_{0}P_{e}L_{n}}{\omega }$$

where $L_{n}$ is the nozzle length, ω is the rotational speed. Actually, the motor rotational speed is the nozzle rotational speed, and the velocity of nozzle ($V_{n}$) can be described by $V_{n}=\omega \times L\;$. We assumed that in Equation (1), the rotational speed could be replaced by the velocity of nozzle, because the centrifugal force and jet velocity is determined by not only rotational speed, but also the turning radius of the nozzle [42]. So, the empirical model can be described by

(2) $$D_{f}\sim \frac{LR_{0}P_{e}L_{n}}{V_{n}}$$

The relationship between final fiber diameter and elastic processability number was described as $D_{f}/R_{0}=0.00162P_{e}^{0.5}$ [39]. After optimization, the final fiber diameter can be described as $D_{f}=0.00162\sqrt{\lambda h_{m}R_{0}}$. Therefore, the empirical model is not an exponential formula, but a power law with exponent ~1/2. Then the empirical model of the fiber diameter could be expressed as

(3) $$D_{f}=k\sqrt{\frac{\lambda h_{m}LR_{0}L_{n}}{V_{n}}}$$

where k is the coefficient of fiber diameter. This empirical model provided a better understanding for nanofiber formation via centrifugal spinning system than any other relation that was studied. Not only intrinsic properties (concentration and viscosity), but also operational conditions (rotational speed, nozzle diameter and nozzle length) were considered into the empirical model to predict the nanofiber formation.

### 3.2. Fabrication of CCS/PEO Nanofibers and Validation of Empirical Model

Different CCS/PEO solutions with different ratios of CCS to PEO were used to test the general relationship between fiber diameter and various parameters. Viscosity was a key parameter for production of continuous bead-free nanofibers [52]. Higher concentration leads to higher viscosity, and, the viscoelasticity of jet increases with viscosity increasing. During jet-extension stage, the solution jet will be stretched by various forces, mainly including centrifugal force, air drag force and viscoelasticity force [42]. If viscosity decreases, while other parameters remains the same, the centrifugal force and air drag force will break-up the jet, due to viscoelasticity effect that cannot overcome the stretching from these external forces. Then, solution jet breakup before jet solidification. Figure 3 shows the corresponding viscosities of different solutions. The CCS solution is very difficult to form nanofibers, due to the viscosity being too low to form sufficient viscoelasticity to withstand the stretch of external force [39], and the CCS chain entanglement being not sufficient to form continuous fibers [38]. Therefore, the concentration of PEO solution in mixed CCS/PEO solution should be higher than the critical concentration (6 w/v%) for bead-free fibers, to improve the viscosities of composite solutions and produce bead-free nanofibers. The rheological behaviors between PEO solutions and CCS solutions were very different, and there was a big gap between 5 w/v% and 6 w/v% PEO solutions (Figure 3). Therefore, the viscosities of CCS/PEO composite solutions combined those of PEO and CCS solutions, and a higher ratio of PEO leads to a higher viscosity. At low shear rate, 0.1–4/s, the viscosities of CCS/PEO solutions were between 5 w/v% and 6 w/v% PEO solutions, while the viscosities of CCS/PEO solutions were higher than 6 w/v% PEO solution after 4/s and 7 w/v% PEO solution after 10/s, respectively. It illustrated that the viscosities of these three CCS composite solutions were high enough to form continuous nanofibers [52]. In addition, Capillary number ($C_{a}$) and Weissenberg number ($W_{i}$) can be used to describe formation of fibers or beads. $C_{a}=t_{vis}/t_{ct}$ and $W_{i}=\;\lambda /t_{ct}$, where $t_{vis}$ is the viscosity time, λ is relaxation time and $t_{ct}$ is the centrifugal time. When $C_{a}>$ 2 and $W_{i}>$ 28, continuous bead-free fibers could be produced [39]. In this centrifugal spinning system, when the rotational speed is over 4000 rpm, these two dimensionless numbers ($C_{a}$ and $W_{i}$) of the three CCS/PEO solutions is around 10 and 140, respectively. Therefore, continuous bead-free nanofibers of CCS/PEO could be fabricated in certain conditions. 

**Figure 4:**
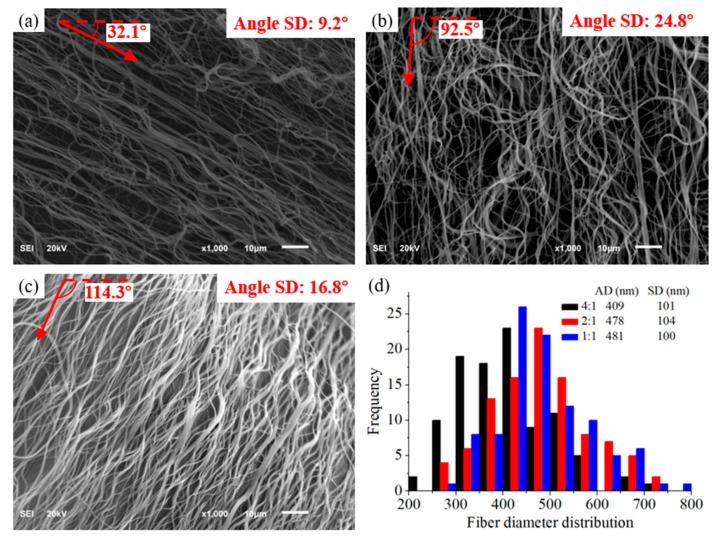
SEM images of nanofibers of different CCS/PEO ratios with corresponding average alignment angles and angle standard deviation (SD). (**a**): 4:1; (**b**); 2:1; (**c**): 1:1; (**d**): fiber diameter distributions of these nanofibers.

In order to produce uniform nanofibers from different solutions and validate the empirical model of Equation (3), fiber diameters of three different ratios of CCS/PEO via setting of various parameters were shown in Table 1. After fabrication of three different CCS/PEO solutions, the SEM images show that the continuous bead-free composite nanofibers were obtained (Figure 4a–c). Besides, the fiber diameter distributions of these CCS/PEO nanofibers (Figure 4d) indicate that the fiber diameter follows empirical model of Equation (3). In addition, all the average fiber diameters (AD) of these nanofibers were in the range of 50–500 nm, and the standard deviation (SD) of these nanofibers illustrated that the nanofibers were uniform, regardless of the ratio of chitosan used in composite nanofibers.

### 3.3. Characterization of Mechanical Properties

**Figure 5:**
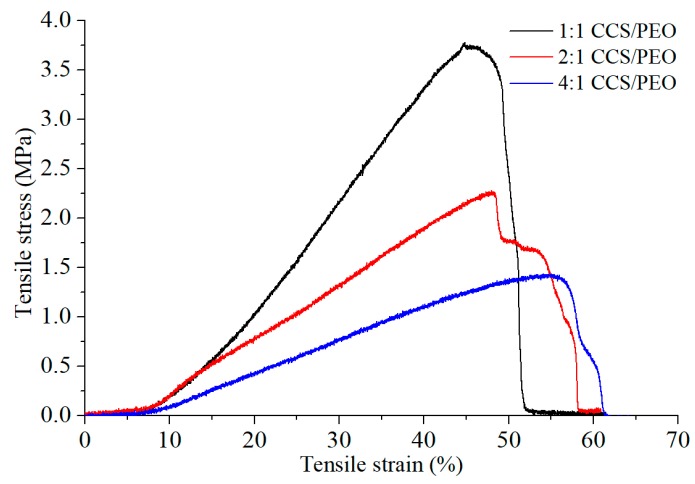
Stress–strain curve of the three different ratios of CCS/PEO nanofiber scaffolds.

Mechanical properties of nanofibers are strongly influenced by many factors, including chemical structure of polymers and physical properties of fibers (diameter distribution, length and entanglement, etc.) [53]. The mechanical properties of the CCS/PEO nanofibers were shown in Figure 5. The results show that the tensile stress decreased (from 3.7 MPa to 1.4 MPa), while the tensile strain gradually increased (from 45% to 55%) with CCS component increasing. Besides, the fiber diameter distribution and the fiber uniformity of the three composite nanofibers are similar. It might illustrate that the tensile strength of PEO was larger than CCS, but the elastic limit of CCS was better than PEO. In tissue regeneration, the cell migration and proliferation in nanofiber scaffolds would be benefited from the ECM mechanical support at the range of 0.8–18 MPa [53,54]. Therefore, the tensile strength of all nanofiber yarns produced from the current study are sufficiently strong for cell culture [55]. Figure 5 also indicated that not all fibers were broken at the same time; instead, a few fibers still remained unbroken after the failure of majority of the fibers. This might attribute to the alignment of 2:1 CCS/PEO (angle SD is 24.8°) which was not as good as others (as shown in Figure 4a–c), because the mechanical properties of nanofibers are also depended on the inherent architecture of the materials [56]. For this reason, the tensile strain of 2:1 CCS/PEO was only slightly larger than 1:1 CCS/PEO nanofiber yarn, and much smaller than 4:1 CCS/PEO nanofiber yarn. In addition, the tensile strain of 4:1 CCS/PEO nanofiber yarn might be larger, if the alignment of the fiber (angle SD is 16.8°) was as good as 1:1 CCS/PEO nanofiber yarn (angle SD is 9.2°). Therefore, higher alignment of fibers might lead to a larger tensile strain.

### 3.4. Thermal Characteristics of CCS/PEO Nanofibers

**Figure 6:**
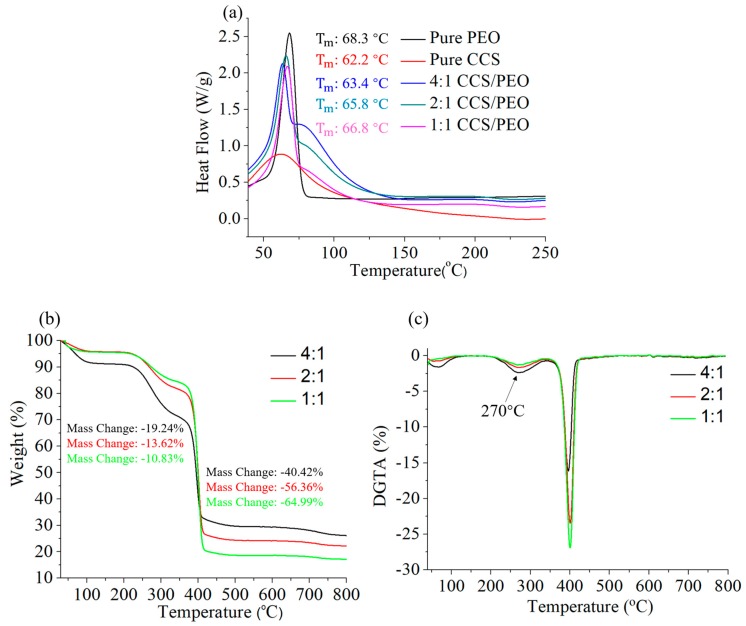
Thermal behaviors of the different nanofibers: DSC (**a**), TGA (**b**) and DTGA (**c**).

Thermal behaviors of nanofibers with different CCS/PEO ratios were shown in Figure 6. It is illustrated that the melting peak points ($T_{m}$) of these materials were similar, even though $T_{m}$ of PEO was slightly higher than that of CCS. In addition, pure PEO powders and CCS/PEO composite nanofibers were much stronger than pure CCS. Specifically, the melting points of these composite nanofibers were between pure PEO and pure CCS, and the value slightly increased with increasing PEO content, from 63.4 °C to 66.8 °C. There was only one melting point for each composite nanofibers, demonstrating that a strong intermolecular hydrogen bond formed between ether groups of PEO and amino/hydroxyl groups of CCS [57,58]. Figure 6b shows the thermogravimetric analyses of CCS/PEO composite nanofiber scaffolds. As previously reported [57,59], a low weight loss occurred at 80–100 °C due to the moisture present in those nanofiber scaffolds, and the higher weight loss of 4:1 CCS/PEO nanofiber scaffold than the others might be because there was more water in the scaffold. At the range of 100–200 °C, the scaffolds were stable and no weight loss. After 200 °C, these scaffolds started losing weight, and until around 340 °C, a large weight loss occurred due to the CCS started degradation while PEO was stable. The largest weight loss occurred at the range of 340–400 °C, because both of CCS and PEO were degraded. When the temperature was over 400 °C, the weight of these nanofiber scaffolds was stable and the degradation was finished. Finally, the higher content of CCS leads to larger weight residue. Figure 6c shows that there are two degradation rate peaks for each sample at 270 °C and 400 °C, respectively. It illustrated that the maximum degradation rate of those CCS/PEO composite nanofibers occurred by compounding of PEO and CCS [59]. Therefore, these nanofiber scaffolds were thermally stable.

### 3.5. Chemical Composition of CCS/PEO Nanofibers

**Figure 7:**
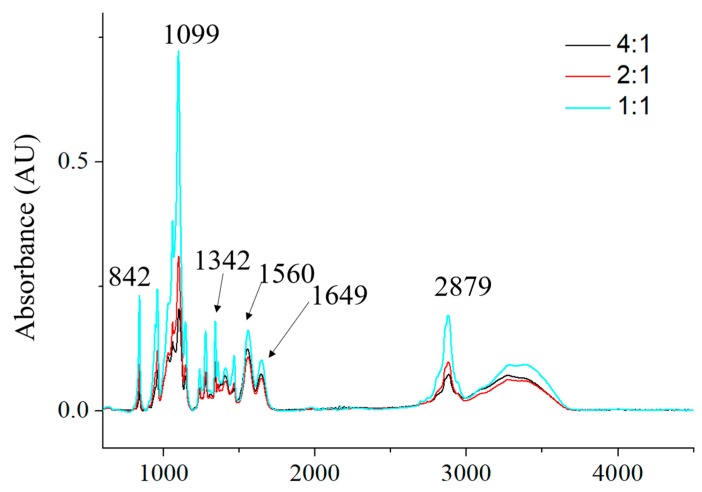
The FTIR-ATR spectra of the three different ratios of CCS/PEO nanofiber scaffolds.

Chemical compositions of the three different CCS/PEO nanofiber scaffolds were characterized by FTIR-ATR spectra, as shown in Figure 7. All characteristic peaks of these three scaffolds were almost the same, which illustrated that the chemical structures of these three scaffolds were the same. The first peak at 842 cm^−1^ was attributed to the C–O stretching on CCS/PEO scaffolds [13]. The peak of 962 cm^−1^ was due to the vibration of ether group [60]. The triplet peaks of 1060, 1099 and 1145 cm^−1^ were attributed to the C–O–C stretching vibrations [57,59]. The peaks of 1342, 1560 and 1649 cm^−1^ were attributed to amide III, amide I and amide II, respectively [13,61]. The peak of 2879 cm^−1^ was due to the CH_2_ stretching [20]. A broad bond at 3200–3400 cm^−1^ was detected due to N–H and O–H stretching of the polysaccharide molecules [12]. These results indicated that the chemical structures of CCS and PEO were maintained with no additional compounds formed after blended fabrication via centrifugal spinning.

## 4. Conclusions 

In this study, we investigated various parameters, including nozzle diameter, rotational speed, concentration and nozzle length, for the fabrication of nanofibers. Based on the obtained nanofibers, more parameters were considered into an empirical model, so as to accurately predict the diameter of produced fibers in this centrifugal spinning system. The physical and chemical properties of CCS/PEO nanofibers were characterized via DSC, TG, FTIR-ATR and mechanical test. In this centrifugal spinning system, higher concentration polymers lead to less solvent, comparing with electrospinning. Therefore, less harmful gas will be produced in the air, if the solvent is toxic or contaminative. Continuous bead-free ultra-fine and uniform nanofibers with high content of chitosan was fabricated to improve antibacterial and biocompatible properties of scaffolds for tissue engineering application. Centrifugal spinning could be an alternative method for production of nanofibers with uniform morphology and high efficiency, so as to incorporate the advantages of needle and needleless electrospinning processes. In vitro and vivo experiments will be further studied to assess the advantages and disadvantages of the produced nanofibers.

## Figures and Tables

**Table 1:** Parameters setting of the three different ratios of CCS/PEO solutions.

CCS:PEO	Rotational Speed (rpm)	Nozzle Diameter (mm)	Nozzle Length (mm)
4:1	4500	0.1	6.5
2:1	4500	0.1	13
1:1	5000	0.1	13
